# A Cluster Randomised Trial of a Multifaceted Quality Improvement Intervention in Brazilian Intensive Care Units

**DOI:** 10.1186/2197-425X-3-S1-A24

**Published:** 2015-10-01

**Authors:** AB Cavalcanti, FA Bozza, FR Machado, JIF Salluh, VP Campagnucci, P Vendramim, HP Guimarães, K Normílio-Silva, VC Chiattone, LP Damiani, ER Romano, F Carrara, J Lubarino, AR Silva, G Viana, C Teixeira, NB Silva, CCH Chang, DC Angus, O Berwanger, The CHECKLIST-ICU Investigators and the Brazilian Research in Intensive Care Network (BRICNet)

**Affiliations:** Hospital do Coração, Research Institute, São Paulo, Brazil; D'Or Institute for Research and Education, Rio de Janeiro, Brazil; Oswaldo Cruz Foundation, Rio de Janeiro, Brazil; Latin American Sepsis Institute, Sao Paulo, Brazil; Hospital Samaritano, Sao Paulo, Brazil; Hospital do Coração, Research Institute, Sao Paulo, Brazil; D'Or Institute for Research and Education, Sao Paulo, Brazil; Hospital Moinhos de Vento, Sao Paulo, Brazil; University of Pittsburgh, Pittsburgh, United States

## Introduction

Checklists, daily goal assessments, and clinician prompts have been proposed as quality improvement (QI) strategies in intensive care units (ICU). However, their effectiveness in improving safety climate, adherence to care processes and clinical outcomes is uncertain.

## Objectives

To evaluate whether the use of a multifaceted QI intervention, including the use of a checklist and the definition of daily care goals during multidisciplinary rounds, and clinician prompts, lowers in-hospital mortality in Brazilian ICUs. Secondary objectives were to assess whether the QI intervention would improve care processes, safety climate and clinical outcomes.

## Methods

We first conducted an observational phase to obtain baseline data on safety culture, care processes and clinical outcomes. Thereafter, we randomly assigned 118 Brazilian ICUs to a QI intervention consisting of a daily checklist and definition of care goals during multidisciplinary rounds with follow-up clinician prompting regarding daily goals, or to routine care. The primary outcome, in-hospital mortality, truncated at 60 days, measured in the first 60 admissions of >48h to each ICU, was analyzed using a random effects logistic regression model, adjusted for patients severity and ICU's baseline standardized mortality ratio. Secondary outcomes included adherence to care processes, ICU safety climate and clinical events.

## Results

We enrolled 13,638 patients in the 118 ICUs, including 6,877 patients in the pre-randomization phase and 6,761 in the randomized phase. Primary outcome data were available for 99.9% of the patients. Adherence to the QI intervention was good. The intervention improved 4 of 7 care processes: increased use of lower tidal volumes (rate ratio [RR] 1.14; 95% confidence interval [CI], 1.03 to 1.26; P = 0.01) and number of days that patients were under light sedation or alert and calm (RR 1.19; 95% CI, 1.00 to 1.42; P = 0.05), and decreased use of central venous (RR 0.90; 95% CI, 0.83 to 0.98; P = 0.02) and urinary catheters (RR 0.86; 95% CI, 0.80 to 0.93; P < 0.01). It also improved teamwork (odds ratio [OR] 1.30; 95% CI, 1.08 to 1.57; P = 0.01) and safety climate (OR 1.27; 95% CI, 1.02 to 1.57; P = 0.03). There were 1,095 in-hospital deaths (32.9%) in the intervention group and 1,196 (34.8%) in the control group (odds ratio 1.02; 95% CI, 0.82 to 1.26; P = 0.88). There were no differences in other clinical outcomes.

## Conclusions

A QI intervention including a checklist and setting of daily goals, and clinician prompting improved care processes and safety climate. However it did not improve in-hospital mortality or other clinical outcomes.

## Grant Acknowledgment

Funded by Brazilian Development and Social Bank and Program to Support Institutional Development of Universal Health System. Registered at ClinicalTrials.gov NCT01785966.

